# Prevalence of SFTSV among Asian House Shrews and Rodents, China, January–August 2013

**DOI:** 10.3201/eid2012.141013

**Published:** 2014-12

**Authors:** Jian-Wei Liu, Hong-Ling Wen, Li-Zhu Fang, Zhen-Tang Zhang, Shu-Ting He, Zai-Feng Xue, Dong-Qiang Ma, Xiao-Shuang Zhang, Tao Wang, Hao Yu, Yan Zhang, Li Zhao, Xue-jie Yu

**Affiliations:** School of Public Health, Shandong University, Jinan, China (J.-W. Liu, H.-L. Wen, L.-Z. Fang, S.-T. He, X.-S. Zhang, L. Zhao, X.-j. Yu);; Huangdao District Center for Disease Control and Prevention, Qingdao City, China (Z.-T. Zhang, Z.-F. Xue, D.-Q. Ma);; Zibo Municipal Center for Disease Control and Prevention, Zibo, China (T. Wang);; University of Texas Medical Branch, Galveston, Texas, USA (H. Yu, X-j. Yu);; College of Medicine and Nursing, Dezhou University, Dezhou City, China (Y. Zhang)

**Keywords:** severe fever with thrombocytopenia syndrome virus, SFTS virus, bunyavirus, seroprevalence, shrews, rodents, viruses, animal hosts, China

## Abstract

To evaluate the role of small mammals as hosts of severe fever with thrombocytopenia syndrome virus (SFTSV), we tested serum samples from rodents and shrews in China, collected in 2013. SFTSV antibodies and RNA were detected, suggesting that rodents and shrews might be hosts for SFTSV.

Severe fever with thrombocytopenia syndrome (SFTS) is an emerging hemorrhagic fever caused by SFTS virus (SFTSV), a recently discovered phlebovirus in the family *Bunyaviridae* (*1*). SFTS has been reported in humans in China, South Korea, and Japan since 2010 (*1*–*3*). During 2011–2012, an outbreak with 2,047 reported SFTS cases occurred in China (*4*). The disease has a high case-fatality rate (12%–30%) (*1*).

SFTSV has been detected in *Haemaphysalis longicornis* ticks, the probable source of transmission to humans (*1*), and person-to-person transmission of SFTSV through contact with an infected person’s blood or mucus has been reported (*5*,*6*). SFTSV has been detected in domestic animals, including goats, cattle, dogs, and chickens (*7*–*10*). Small mammals, such as rodents and shrews, are the major hosts of *H. longicornis* tick larvae and nymphs (*11*); however, the role of small mammals as hosts of SFTSV is not well defined. In this study, we determined the seroprevalence of SFTSV and the prevalence of SFTSV viral RNA among rodents and shrews captured in rural areas of eastern China.

## The Study

During January–August 2013, we collected rodents and shrews in Jiaonan County, Shandong Province, China (119°30′–120°30′E, 35°35′–36°08′ N). The animals were trapped once a month by using mouse traps baited with peanuts; a different trap site was used each month. The traps were set before sunset outside human dwellings in rural areas and collected the next morning. We classified the rodents and shrews according to appearance (hair color) and body structures (i.e., shape of mouth, teeth, tail, ratio of tail length to body length, cheek pouch). After the animals were euthanized, we collected blood samples from their hearts onto absorbent paper strips (10 mm × 30 mm). Spleens were collected aseptically and frozen at −80°C. Animal use and sample collection protocols were approved by the bioethics committee of the School of Public Health, Shandong University.

Before testing the serum samples for the presence of SFTSV, we evaluated the sensitivity and specificity of a double-antigen sandwich ELISA kit (Wuxi Xinlianxin Biotech Co, Wuxi City, China) for detecting SFTSV in rodent serum. To do this, we used an indirect immunofluorescence assay (IFA) as a standard to test rodent serum samples for SFTSV and compared the results with those from the ELISA. A total of 68 Kunming albino mice (Shandong University Experimental Animal Center, Jinan, China) were used for the tests: 36 were injected with SFTSV, and 32 (controls) were injected intraperitoneally with saline solution. On days 7, 12, 17, and 21 after injection, 9 infected and 8 control mice were euthanized, and serum samples from the mice were tested for SFTSV antibody by IFA and ELISA. Of the 36 serum samples from the SFTSV-inoculated mice, 33 were positive for SFTSV by IFA and ELISA and 3 were positive by IFA only. All 32 serum samples from the control mice were negative for SFTSV by IFA and ELISA. Thus, under these laboratory conditions and compared with the IFA, the ELISA had a sensitivity of 91.7% and specificity of 100%, suggesting that the ELISA could be used to test serum samples from the field-collected rodents. Kits for shrews were not commercially available in China, so we were unable to test the sensitivity and specificity of the ELISA for detecting SFTSV in serum from shrews.

We collected 89 Asian house shrews (*Suncus murinus*) and 666 rodents in the field during January–August 2013. The rodents included 186 striped field mice (*Apodemus agrarius*), 182 house mice (*Mus musculus*), 156 brown rats (*Rattus norvegicus*), 125 greater long-tailed hamsters (*Cricetulus tyiton*), and 17 Chinese hamsters (*Cricetulus barabensis*). Serum samples from the rodents and shrews were tested for IgG and IgM to SFTSV by using the ELISA as described previously (*8*–*10*). Serum samples on absorbent paper were each reconstituted by adding 150 μL of phosphate-buffered saline, and 75 μL of each sample was added to a well of the ELISA plate.

ELISA results showed that SFTSV seroprevalence was higher among Asian house shrews (4.5%, 4/89) than among rodents (0.9%, 6/666) (p = 0.014). Among the rodents, SFTSV seropositivity was higher in house mice (1.1%, 2/182) and striped field mice (1.1%, 2/186) than in other rodent species (Table). However, the seropositivity rate was not significantly different among the rodent species (p = 0.691). In addition, the seropositivity rate did not differ significantly by month (p = 0.411). Statistical analyses were performed by using the Fisher exact test.

**Table:** Severe fever with thrombocytopenia syndrome virus seroprevalence rate and PCR positivity rate among rodents and shrews, Jiaonan County, Shandong Province, China, January–August 2013

Animal	No. seropositive/no. total (%)	No. PCR positive/no. total (%)	
*Mus musculus* mice	2/182 (1.1)	1/103 (1.0)	
*Rattus norvegicus* rats	0/156	1/116 (0.9)	
*Cricetulus tyiton* hamsters	1/125 (0.8)	1/83 (1.2)	
*Apodemus agrarius* mice	2/186 (1.1)	0/129	
*Cricetulus barabensis* hamsters	0/17	0/9	
*Suncus murinus* shrews	4/89 (4.5)	2/77 (2.6)
Total	9/755 (1.2)	5/517 (1.0)

Total RNA was extracted from homogenized animal spleens by using the RNeasy Mini Kit (QIAGEN, Hilden, Germany), which was used as a template for SFTSV amplification performed by using the Access RT-PCR System (Promega, Madison, WI, USA). Primers for reverse transcription PCR (RT-PCR) were designed from the M segments of the SFTSV genome. Outside PCR primers were 5′-TCTGCAGTTCAGACTCAGGGA-3′ and 5′-GACGTGTATTGCTGTTTTCCC-3′; nested PCR primers were 5′-TGTTGCTTGTCAGCCTATGAC-3′ and 5′-CAACCAATGATCCTGAGTGGA-3′. The PCR products (674 bp) were cloned and sequenced for both strands at least 3 times. Amplification results showed that the SFTSV positivity rate was higher among shrews (2.6%, 2/77) than among rodents (0.7%, 3/440), but the difference was not significant (p = 0.162) (Table). The PCR positivity rate differed by month: the rate was 4.7% (4/86) for animals trapped in March, 1.6% (1/62) for animals trapped in June, and 0 for animals collected during the other 6 months (p = 0.043).

Phylogenetic analysis indicated that the SFTSV sequences from the rodents and shrews were closely related to each other (98.5%– 99.7% homology) and highly homologous (95.3% to 99.7%) to the corresponding sequences of SFTSV from a human (GenBank accession no. KC505127), a dog (GenBank accession no. JF267784), and ticks (GenBank accession nos. KC473541 and JQ684872) (Figure). DNA sequences were deposited in GenBank (accession nos. KF770995–9).

**Figure:**
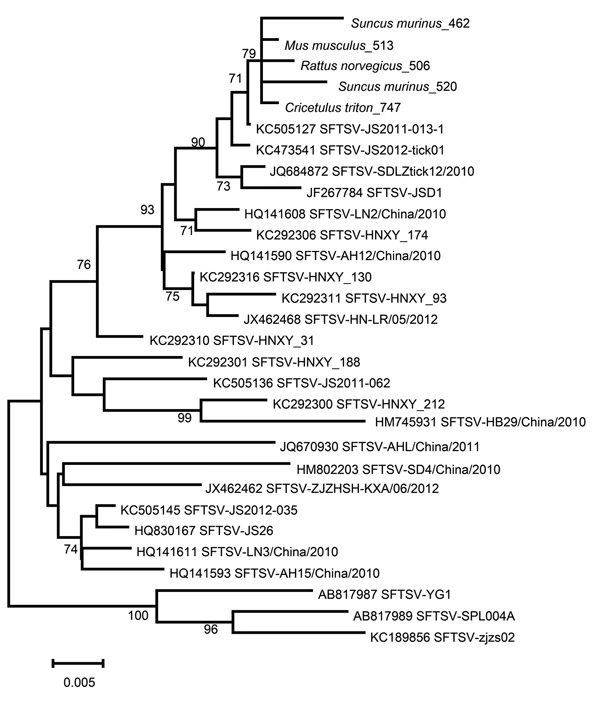
Phylogenetic analysis of severe fever with thrombocytopenia syndrome virus (SFTSV) amplified from the spleens of Asian house shrew and rodents. The neighbor-joining phylogenetic tree was constructed by using MEGA 5.2 software(http://www.megasoftware.net/).GenBank accession numbers precede isolate names on the right side of the figure. Numbers at nodes represent bootstrap values. Scale bar represents nucleotide substitutions per site.

## Conclusions

Two previous studies demonstrated that rodents in China were seronegative for SFTSV (*7*,*12*). However, negative results might have been caused by small sample sizes or by using rodents that were collected from sites where SFTSV was not endemic. The serologic and RT-PCR results in our study show that rodents and shrews in eastern China are seropositive for SFTSV. These findings suggest that these animals are potential hosts for SFTSV. We also attempted to amplify S segments of the genomes of SFTSV isolates, but results were positive for only 1 animal. It is possible that in our study, the primer for the S segment was not as sensitive as that for the M segment.

Most animal species that have been investigated for the presence of SFTSV have been positive for the virus, including rodents, shrews, goats, cattle, dogs, and chickens (*5*,*8*–*10*). Thus, there may be numerous animal hosts for SFTSV. Goats have usually shown high seroprevalence to SFTSV but very low virus loads and short periods of SFTSV viremia (*9*). Rodents experimentally infected with SFTSV usually have long periods of viremia and high virus loads in the blood (*13*,*14*).

Cross-sectional analysis indicated that the SFTSV infection rate among rodents and shrews did not differ substantially by month during the 8-month study period, but the PCR positivity rate was highest for animals collected during March. The variation between months can probably be explained by the fact that the animals were collected from different sites each month; the rates of infection among animals at the different sites may have differed.

In conclusion, our findings show that the Asian house shrew and different varieties of rodents are potential animal hosts of SFTSV. Further studies are needed to determine which of the many animal hosts of SFTSV can most effectively transmit the virus to ticks, the probable vector of SFTSV transmission to humans.
